# shRNA transgenic swine display resistance to infection with the foot-and-mouth disease virus

**DOI:** 10.1038/s41598-021-95853-3

**Published:** 2021-08-12

**Authors:** Wenping Hu, Haixue Zheng, Qiuyan Li, Yuhang Wang, Xiangtao Liu, Xiaoxiang Hu, Wenjie Liu, Shen Liu, Zhisheng Chen, Wenhai Feng, Xuepeng Cai, Ning Li

**Affiliations:** 1 State Key Laboratory of AgroBiotechnology, China Agricultural University, Beijing, China; 2 State Key Laboratory of Veterinary Etiological Biology, National Foot and Mouth Diseases Reference Laboratory, Lanzhou Veterinarian Research Institute, Chinese Academy of Agricultural Sciences, Lanzhou, Gansu China; 3 Key Laboratory of Animal Genetics and Breeding and Reproduction of Ministry of Agriculture and Rural Affairs, Institute of Animal Sciences, Chinese Academy of Agricultural Sciences, Beijing, 100193 China; 4 Beijing Genprotein Biotechnology Company, Beijing, China

**Keywords:** Cloning, Genetic engineering

## Abstract

Foot-and-mouth disease virus (FMDV) is one of the most important animal pathogens in the world. FMDV naturally infects swine, cattle, and other cloven-hoofed animals. FMD is not adequately controlled by vaccination. An alternative strategy is to develop swine that are genetically resistant to infection. Here, we generated FMDV-specific shRNA transgenic cells targeting either nonstructural protein 2B or polymerase 3D of FMDV. The shRNA-positive transgenic cells displayed significantly lower viral production than that of the control cells after infection with FMDV (*P* < 0.05). Twenty-three transgenic cloned swine (TGCS) and nine non-transgenic cloned swine (Non-TGCS) were produced by somatic cell nuclear transfer (SCNT). In the FMDV challenge study, one TGCS was completely protected, no clinical signs, no viremia and no viral RNA in the tissues, no non-structural antibody response, another one TGCS swine recovered after showing clinical signs for two days, whereas all of the normal control swine (NS) and Non-TGCS developed typical clinical signs, viremia and viral RNA was determined in the tissues, the non-structural antibody was determined, and one Non-TGCS swine died. The viral RNA load in the blood and tissues of the TGCS was reduced in both challenge doses. These results indicated that the TGCS displayed resistance to the FMDV infection. Immune cells, including CD3^+^, CD4^+^, CD8^+^, CD21^+^, and CD172^+^ cells, and the production of IFN-γ were analyzed, there were no significant differences observed between the TGCS and NS or Non-TGCS, suggesting that the FMDV resistance may be mainly derived from the RNAi-based antiviral pathway. Our work provides a foundation for a breeding approach to preventing infectious disease in swine.

## Introduction

Foot-and-mouth disease (FMD) is a highly contagious disease that affects more than 70 species of domestic and wild cloven-hoofed animals, including swine, cattle, goat, sheep, buffalo, and deer^1^. As an acute disease, FMD has a variety of symptoms, characterized by fever, nasal discharge, lameness, and vesicular lesions on the muzzle, tongue, teats, and feet^2,3^, and it is lethal in young animals. Foot-and-mouth disease virus (FMDV) is the pathogen responsible for FMD. FMDV rapidly replicates in infected animals and transmits through contact with susceptible animals and by aerosol^1^. FMD causes enormous global economic losses for livestock production and trade because in addition to the slaughter of millions of animals, many countries refuse to trade livestock with countries that have known FMD epidemics^4^.

FMDV is an RNA virus belonging to the Aphthovirus genus of the Picornaviridae family^5,6^, and its genome is a single-coding, positive-sense RNA of approximately 8500 nt in length^1, 7^. FMDV has seven distinct serotypes (A, O, C, SAT1, SAT2, SAT3, and Asia 1) and many subtypes in each serotype^5,8–10^. The diversity and potential for evolutionary shift and drift of FMDV are a challenge. Current vaccines just provide no clinical symptoms, are unable to prevent viral replication, or eliminate the development of viral carriers in animals that have been vaccinated. One novel alternative strategy to control FMDVs in livestock is to introduce novel genes that provide resistance to FMDVs.

Fire et al. described a process found in *Caenorhabditis elegans*^11^, called RNA interference (RNAi), a naturally occurring process triggered by a double-stranded RNA (dsRNA) or short hairpin RNA (shRNA) that can be processed into small interfering RNAs (siRNAs), interact with a protein complex, and cleave their complementary RNAs^12, 13^. RNAi reduces gene expression through mRNA degradation, the repression of transcription, and the inhibition of translation^14^. These processes were also observed in plants, insects, nematodes, and fungi as a natural antiviral defense^15–17^. Recently, the antiviral RNAi pathway in mammalian cells has been reported^17,18^.

Many human viruses have been successfully targeted by RNAi, such as human pathogen respiratory syncytial virus (RSV)^19^, hepatitis B virus (HBV)^20–22^, hepatitis C virus (HCV)^23–25^, human immunodeficiency virus type 1(HIV-1)^26–31^, severe acute respiratory syndrome coronavirus (SARS-CoV)^32,33^, yellow fever virus^34^, and influenza A virus^35,36^. Some compounds are currently being tested in clinical trials^37^. In addition, respiratory viruses^38^ and herpes simplex virus 2^39^ in mice; porcine reproductive and respiratory syndrome virus^40^, porcine endogenous retrovirus^41^, and porcine circovirus type 2^42^ in swine; and Marek’s disease virus^43^ in chickens were targeted by RNAi.

Several studies have been conducted on the inhibition of FMDV replication by siRNAs in vitro^44–48^. RNAi delivered by adenovirus or an attenuated Salmonella choleraesuis expression vector (shRNA) reduced the susceptibility of porcine IBRS-2 cell guinea pigs and swine to FMDV infection^7,49^. The data obtained in cell culture and suckling mouse assays demonstrated that siRNAs targeting conserved regions of the FMDV genome could be effective against FMDV. The potential of RNAi as an antiviral strategy against FMDV in relevant animal systems is of great interest, and the generation of a novel swine breed harboring the shRNA transgene may be an alternative approach for controlling FMD.

Here, we constructed an shRNA-expressing vector targeting conserved sequences within the coding regions of viral polymerase protein 3D and nonstructural protein 2B of the FMDV genome, which has the potential to interfere with the virus replication and packaging. Then, we generated transgenic cloned swine and challenged them with FMDV, with Non-transgenic cloned swine as a control. The transgenic cloned swine displayed resistance to the FMDV infection, and the virus level in the blood and tissues was dramatically decreased, indicating that the shRNA transgenic swine might prevent virus transmission and control the outbreak of FMDV epidemics. The results provide insight into the use of RNAi in animal breeding for disease resistance.

## Results

### shRNA transgenic cells exhibited the efficient anti-FMDV ability

The shRNA expression vector pMD19-3D-EGFP-NEO-2B (p3DEN2B) was constructed to target either nonstructural protein 2B or polymerase 3D of FMDV and expressed enhanced green fluorescent protein (EGFP) and Neo-R (Supplementary Fig. S1A). The 2B-shRNA was 25 nt (nucleotides), and the 3D-shRNA was 56 nt. We constructed a control vector, pMD19-EGFP-NEO (pEN), which did not express shRNAs but expressed EGFP and Neo-R (Supplementary Fig. S1B). Baby hamster kidney cells (BHK-21) and swine kidney cells (IBRS-2) were used to examine the antiviral activity. Four transgenic cell lines were generated: (1) BHK-21-positive transgenic cells that contain p3DEN2B (BHK21-P), (2) BHK-21-negative transgenic cells that contain pEN (BHK21-N), (3) IBRS-2-positive transgenic cells that contain p3DEN2B (IBRS2-P), and (4) IBRS-2-negative transgenic cells that contain pEN (IBRS2-N). The FMDV strain O/Guangdong/CHA/86 was used in all of the infection and challenge studies.

The siRNA expression in the four cell lines was confirmed by real-time RT-PCR (Supplementary Fig. S2). The cells were infected with FMDV at a dose of 100 or 1000 TCID_50_, and the total small RNAs were extracted at 0 and 12 h post-infection (h.p.i.). siRNA expression in the BHK21-P and IBRS2-P cells was stable, and there was no difference between 0 h.p.i. and 12 h.p.i. at the doses of 10 and 100 TCID_50_. However, there was a significant difference between the BHK21-P and BHK21-N cells (*P* < 0.001) and between IBRS2-P and IBRS2-N cells (*P* < 0.001) at both doses (Supplementary Fig. S2).

**Figure 1:**
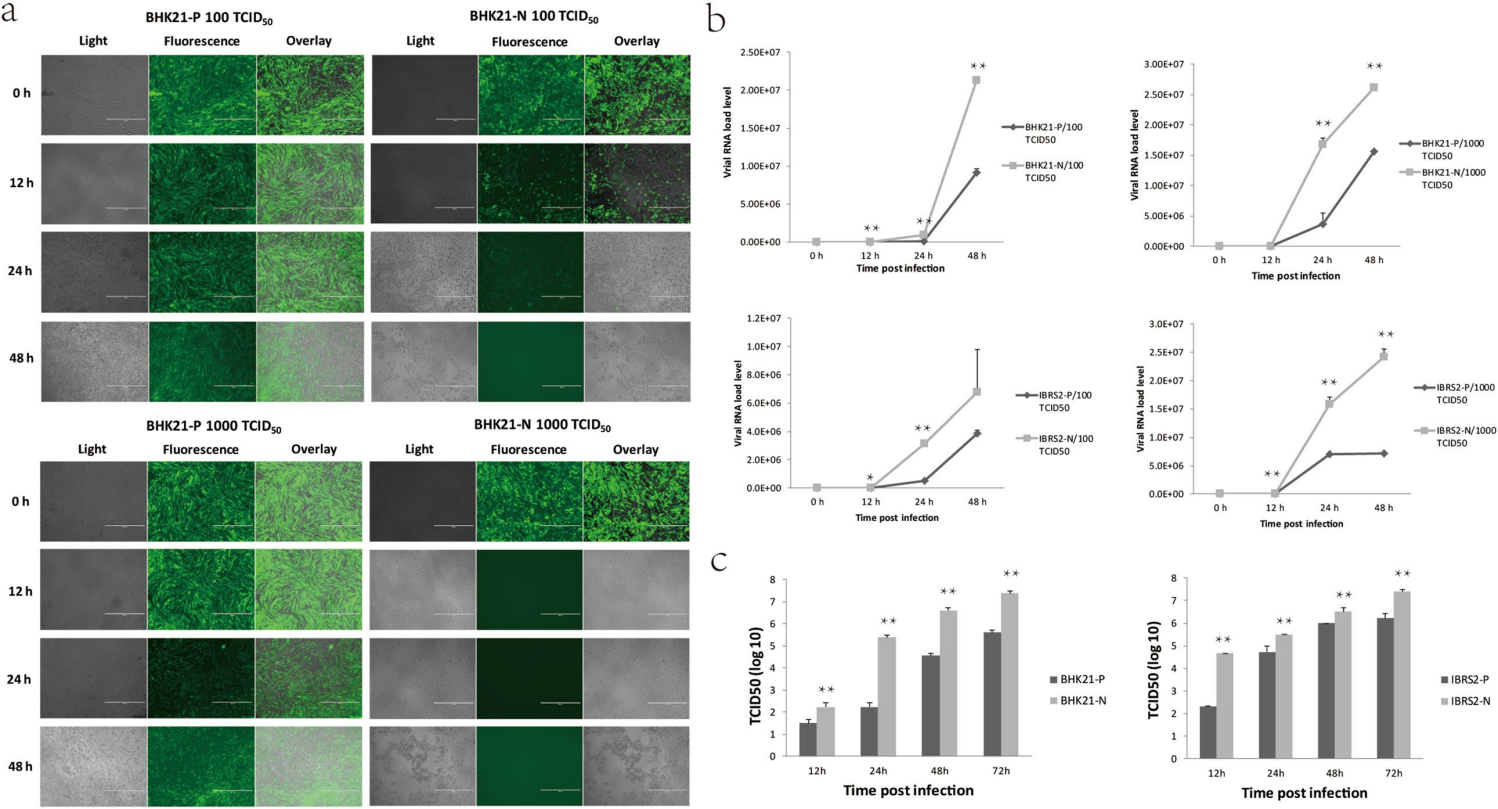
shRNA expression in the BHK-21 and IBRS-2 cells conferred resistance to FMDV infection. (**a**) The BHK21-P, BHK21-N, IBRS2-P and IBRS2-N cell lines were challenged with 100 or 1000 TCID_50_ of FMDV. All four cell lines can express GFP. The cytopathic effect (CPE) was observed at 0, 12, 24 and 48 h.p.i. under a microscope able to show the image under normal white light (Light) and green fluorescence (Fluorescence) as well as the overlay of these two images (Overlay). (**b**) The cell samples were collected at 0, 12, 24, and 48 h.p.i., and the viral RNA load was detected by real-time RT-PCR. The viral replication dynamics in the BHK-21 and IBRS-2 cell lines at various time points are shown here. (**c**) The viral production (TCID_50_) results showed that shRNA expression protected the transgene-positive cell lines from FMDV infection. The BHK-21 cell lines and IBRS-2 cell lines were challenged with 100 TCID_50_ of FMDV. The culture supernatants were collected at 12, 24, 48, and 72 h.p.i., and the virus titer, TCID_50_, was measured using the Reed-Muench formula. The error bars represent the standard error. *Indicates a *P*-value ≤ 0.05; **Indicates a *P*-value ≤ 0.01. *BHK21-P* the BHK-21-positive transgenic cells that contain the p3DEN2B shRNA expression vector, *BHK21-N* the BHK-21-negative transgenic cells that contain the pEN control vector that does not express the shRNA, *IBRS2-P* the IBRS-2-positive transgenic cells that contain the p3DEN2B vector, *IBRS2-N* the IBRS-2-negative transgenic cells that contain the pEN vector.

The cytopathic effect (CPE) and viral RNA load were detected to confirm the anti-FMDV response. Cells were seeded in 12-well plates at the same time and infected with FMDV at a dose of 100 or 1000 TCID_50_. One hour later, the inoculum was removed and fresh medium was added. The cells were observed at 0, 12, 24 and 48 h.p.i. under a fluorescence microscope. At 0 h, more than 95% cells of each of the four cell lines expressed GFP and no CPE was observed (Fig. 1). At the other time points, the BHK21-P or IBRS2-P cells displayed better antiviral capacity at both infection doses compared to the BHK21-N or IBRS2-N cells. The BHK21-N cells infected with 100 TCID_50_ of the virus exhibited severe CPE at 12 h.p.i. and had all died at 24 h.p.i. However, the BHK21-P cells showed significant resistance to FMDV. No CPE was observed at 0–24 h.p.i., and only mild CPE was observed at 48 h.p.i. (Fig. 1a). The BHK21-N cells infected with 1000 TCID_50_ of the virus had all died at 12 h.p.i., whereas the BHK21-P cells showed resistance to FMDV, with only slight CPE at 24 and 48 h.p.i. (Fig. 1a). Similar results were observed in the IBRS-2 cell lines (Supplementary Fig. S3).

The cell culture samples were collected at every time point, and the viral replication dynamics (Fig. 1b) were determined by one-step real-time quantitative RT-PCR (one-step qRT-PCR). The viral RNA load was still very low at 12 h.p.i. , about 10^2^ to 10^5^, and it had reached 10^7^ at 24 and 48 h.p.i. The BHK21-P cells had a significantly lower viral RNA load than the BHK21-N cells at all of the selected time points after infection with 100 TCID_50_ FMDV (*P* < 0.001). The BHK21-P cells had a significantly lower viral RNA load than the BHK21-N cells at 24 and 48 h.p.i. (*P* < 0.01) after infection with 1000 TCID_50_, although this difference was not significant at 12 h.p.i. (*P* = 0.06). Similar results were determined in the sample of the IBRS-2 cell lines (Fig. 1b).

Viral production was also analyzed in the cells after infection to determine the resistance to FMDV. In the viral infection study, 100 TCID_50_ of FMDV was used to infect the cells, which were seeded in a 96-well plate. The supernatants were collected at 12, 24, 48, and 72 h.p.i., and the viral titers were determined. The FMDV-specific shRNA-expression vector induced antiviral effects toward FMDV in both the BHK-21 and IBRS-2 transgenic cells. The viral titers in the supernatants of the BHK21-P cells were reduced by 81% at 12 h.p.i. and by ~ 100% at 24, 48, and 72 h.p.i. compared to the BHK21-N cells and showed significant differences at all of the time points (*P* < 0.01) (Fig. 1c). Similar results were determined in the IBRS-2 cell lines (Fig. 1c).

### Generation of transgenic cloned swine and quantification of shRNA expression in swine

**Figure 2:**
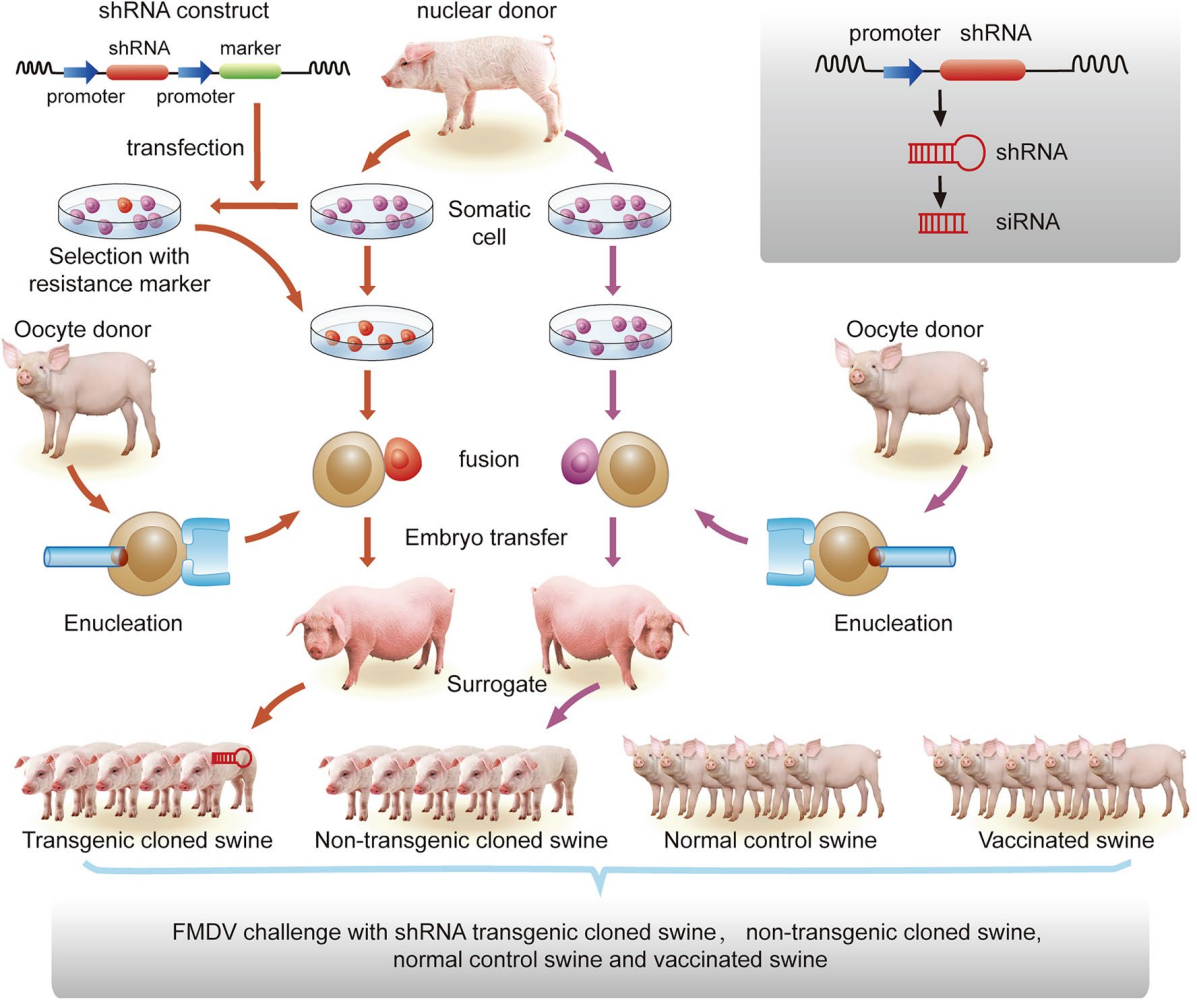
Production of transgenic cloned swine and Non-transgenic cloned swine by SCNT. A male Landrace swine was used as a nuclear donor, and an embryonic fibroblast cell line was established. In the left flow (orange), the shRNA construct was transfected into the embryonic fibroblasts and selected with a resistance marker (the real and clear vector was shown in Supplementary Fig. S1, expressing the 3D-shRNA, EGFP, Neo-R, and 2B-shRNA). The transgene-positive fibroblasts were fused with an enucleated oocyte. The reconstructed embryo was transferred to a surrogate, and the transgenic cloned swine were born. In the right flow (purple), the embryonic fibroblasts were directly used as the nuclear donor cell and fused with an enucleated oocyte. The Non-transgenic cloned swine were born. Then, to determine the anti-FMDV response of the shRNA transgenic cloned swine, we used similar Landrace animals as the Normal control swine and Vaccinated swine to perform the FMDV challenge experiments.

The somatic cells were obtained from the nuclear donor. Some of the somatic cells were transfected with the linearized shRNA expression vector p3DEN2B. The positive transgenic cells were selected with EGFP and the resistance marker Neo-R and were used to produce transgenic cloned swine by somatic cell nuclear transfer (SCNT). The other portion of the somatic cells from the same nuclear donor was used to produce Non-transgenic cloned swine by SCNT (Fig. 2). Thirty-two cloned swine were born, of which 23 positive transgenic cloned swine (19 were alive) and 9 non-transgenic cloned swine (five were alive) were confirmed. The transgenic cloned swine and Non-transgenic cloned swine were cloned from the same somatic cell line and shared the same genome, except for the transgene. The siRNA expression in the transgenic cloned swine was detected by one step qRT-PCR. In the five tested tissues, including the heart, skin, lung, submaxillary lymph node, and kidney, all of the TGCS had a detectable siRNAs expression. The siRNA expression level was approximately 1.1 in the kidney and approximately 0.02–0.08 in the other 4 tissues, with the U6 RNA used as an internal control (Supplementary Fig. S4).

### The clinical symptoms of FMD in transgenic cloned swine were significantly delayed

The same FMDV strain, O/Guangdong/CHA/86, was used to challenge the swine to evaluate the susceptibility of the transgenic swine to FMDV infection. Transgenic cloned swine, Non-transgenic cloned swine, normal control swine, and vaccinated swine were equally separated into two virus challenge experiments. The commercial inactivated FMDV type O vaccine was used to immunize the control “vaccinated swine.” In swine challenge study 1 (the infectious dose was 100 SID_50_), fifteen swine were divided into three groups of five animals per group: (1) the Transgenic cloned swine group (TGCS), (2) Normal control swine group (NS), and (3) Vaccinated swine group (VS). In swine challenge study 2 (infectious dose was 10 SID_50_), fifteen swine were divided into three groups of five animals per group: (1) the Transgenic cloned swine group (TGCS), (2) Non-transgenic cloned swine group (Non-TGCS), and (3) Vaccinated swine group (VS).

**Figure 3:**
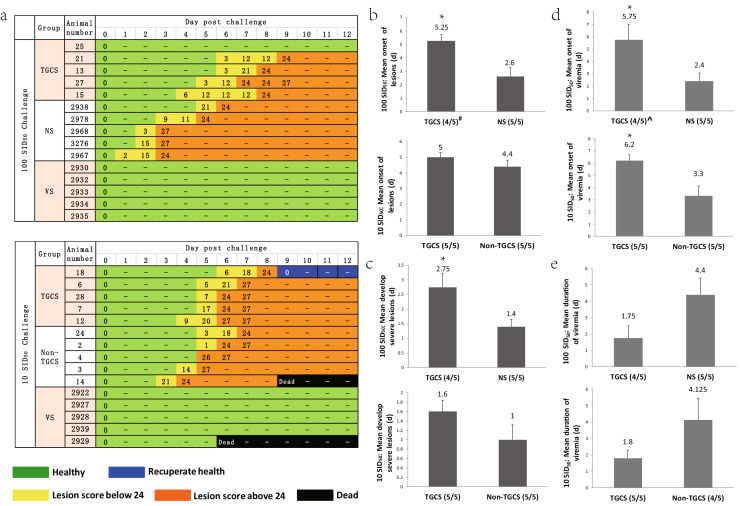
Transgenic cloned swine exhibited delayed clinical symptoms, reduced viremia and displayed resistance to FMDV. *TGCS* transgenic cloned swine group, *Non-TGCS* non-transgenic cloned swine group, *NS* normal control swine group, *VS* vaccinated swine group. (**a**) The development of clinical symptoms and clinical lesion score. Green: healthy; yellow: ill (score below 24); orange: seriously ill (score above 24); black: dead; blue: recuperated and healthy. The number in the column for days post-challenge represents the clinical score, “-” indicates that the clinical score was the same as the last time point. (**b**) Mean onset of lesions (day) in the unprotected swine. (**c**) Mean time of developing severe lesions (days) in the unprotected swine. (**d**) Mean onset of viremia in the unprotected swine (**d**). (**e**) Mean duration of viremia in the unprotected swine. ^#^Swine developed lesions/Swine challenged with virus. ^Swine developed viremia/Swine challenged with virus. *indicates a *P*-value ≤ 0.05. The error bars represent the standard error.

The swine were observed from 0 to 17 days post-challenge (d.p.c.) for clinical symptoms. In the 100 SID_50_ FMDV dose challenge study, one TGCS swine (#25) was completely protected during the experimental period, whereas all five NS swine developed typical FMD clinical signals (Fig. 3a). The other four TGCS also showed a better anti-FMDV response than the NS. The mean onset of the lesions in the other four TGCS swine was delayed by 2.65 days (d) (101.9% later than NS), which was significantly different (*P* < 0.05) (Fig. 3b). The mean time of developing severe lesions for the other infected TGCS was 1.35 d longer than that of the NS (delayed 96.4%), and the difference was also significant (*P* < 0.05) (Fig. 3c). The time of developing severe lesions is the period between the onset of the lesions and the point at which the animals developed the most severe lesions (a lesion score above 24). All five VS swine were protected and no clinical signals (Fig. 3a).

In the 10 SID_50_ FMDV dose challenge study, the TGCS also showed a better anti-FMDV effect than the Non-TGCS, and they performed better in delaying clinical signals. One TGCS swine (#18) had a later onset of lesions by 6 d.p.c., but the lesions were gone by 9 d.p.c., and it completely recuperated (Fig. 3a). The other four TGCS and all of the Non-TGCS developed clinical signs, but one Non-TGCS (#14) died by 9 d.p.c. and none of the sick swine recovered (Fig. 3a). The mean onset of the lesions in the TGCS (including the recuperated swine) was 0.6 d later than that of NS and was delayed 13.6% (Fig. 3b). The mean time of developing severe lesions in TGCS was 0.6 d longer than that in NS a delay of 60% (Fig. 3c). For the VS swine, four VS were protected, and one swine (#2929) died by 6 d.p.c. (Fig. 3a).

The rectal temperature of the swine was monitored twice per day during the experimental period. The mean rectal temperature of the challenged swine showed a similar pattern of changes in the clinical symptoms (Supplementary Fig. S5).

### The FMDV viral RNA load was significantly reduced in the TGCS blood and tissues

RNA was extracted from serum samples collected from 0 to 17 d.p.c., and one-step qRT-PCR was used to determine the viral RNA load. In the 100 SID_50_ FMDV dose challenge study, the viral RNA was not detected in swine #25 in the TGCS group during the whole post-challenge period (Supplementary Table S1A), and this swine remained healthy (Fig. 3a). The viral RNA was detected in the serum samples collected at 7 d.p.c. in the other three swine in the TGCS group but not in the serum samples collected at the other time points (Supplementary Table S1A). However, all five NS were viremic. The mean onset (in days) of viremia in the four viremic TGCS swine was 3.35 d later than that of the five NS (delayed 139.6%), and the difference was significant (*P* < 0.05) (Fig. 3d). The mean duration (in days) of viremia of the four TGCS was 2.65 d (60.2%) shorter than that of the five NS (Fig. 3e). Viremia was not observed in the VS (Supplementary Table S1A). In the 10 SID_50_ FMDV dose challenge study, all five TGCS and Non-TGCS developed viremia (Supplementary Table S1B). However, the mean onset of viremia of the TGCS was delayed by 2.9 d (87.8%) compared to the Non-TGCS, and the difference was significant (*P* < 0.05) (Fig. 3d). The mean duration of viremia of the TGCS was 2.325 d (56.4%) shorter than that of Non-TGCS (Fig. 3e). Viremia was not observed in the VS (Supplementary Table S1B). These results suggested that the TGCS developed viremia much slower and cleared the virus in the blood faster than the NS and Non-TGCS, implying resistance to the FMDV infection.

At day 17 post-challenge, all of the swine were necropsied. Tissue samples were collected for viral RNA load detection. In the 100 SID_50_ FMDV dose challenge study, the FMDV viral RNA was detected in the submaxillary lymph nodes from swine #21 in the TGCS group but not in the submaxillary lymph nodes from the other four TGCS (including swine #25). However, all five NS expressed the viral RNA: two in the spleen, two in the tonsil, and one in the submaxillary lymph nodes (Table 1a). Two VS were randomly selected to detect the tissue viral RNA load, and one of them (#2935) expressed the viral RNA in the kidney and submaxillary lymph nodes. After vaccination, the 10 VS were the ones who already had an antibody response. After the challenge, #2935 exhibited a rapid structural protein/antibody response (data not shown), but the viral RNA was still detected in two tissue types in this animal (Table 1a). In the 10 SID_50_ FMDV dose challenge study, one swine (#12) in the TGCS group expressed viral RNA in the heart and submaxillary lymph nodes, and the other four TGCS did not express viral RNA (Table 1b). Viral RNA was detected in three of the Non-TGCS swine in the lung, submaxillary lymph nodes, tonsil, and mesenteric lymph nodes. Swine #2 expressed viral RNA in all of the tissue types. There was no viral RNA detected in the two randomly selected VS (Table 1b).

**Table 1:** The viral RNA load was reduced in the TGCS tissues after necropsy.

Group	Animal number	Viral RNA copies in tissue samples (100 SID_50_ FMDV challenge)							
		Heart	Liver	Spleen	Lung	Kidney	Submaxillary lymph nodes	Tonsil	Mesenteric lymph nodes
**a**									
TGCS	25	–	–	–	–	–		–	–
TGCS	21	–	–	–	–	–	132.5	–	–
TGCS	13	–	–	–	–	–	–	–	–
TGCS	27	–	–	–	–	–	–	–	–
TGCS	15	–	–	–	–	–	–	–	–
NS	2938	–	–	–	–	–	120.6	–	–
NS	2978	–	–	165.6	–	–	–	–	–
NS	2968	–	–	–	–	–	–	405.8	–
NS	3276	–	–	322.7	–	–	–	–	–
NS	2967	–	–	–	–	–	–	148.6	–
VS	2930	*	*	*	*	*	*	*	*
VS	2932	–	–	–	–	–	–	–	–
VS	2933	*	*	*	*	*	*	*	*
VS	2934	*	*	*	*	*	*	*	*
VS	2935	–	–	–	–	1562.4	553.3	–	–

Group	Animal number	Viral RNA copies in tissue samples (10 SID_50_ FMDV challenge)							
		Heart	Liver	Spleen	Lung	Kidney	Submaxillary lymph nodes	Tonsil	Mesenteric lymph nodes
**b**									
TGCS	18	–	–	–	–	–	–	–	–
TGCS	6	–	–	–	–	–	–	–	–
TGCS	28	–	–	–	–	–	–	–	–
TGCS	7	–	–	–	–	–	–	–	–
TGCS	12	137.1	–	–	–	–	366.8	–	–
Non-TGCS	24	–	–	–	–	–	112	–	–
Non-TGCS	2	–	–	–	108.3	–	320.5	269	148.6
Non-TGCS	4	–	–	–	–	–	–	–	–
Non-TGCS	3	–	–	–	–	–	–	–	–
Non-TGCS	14	–	–	–	–	–	153.7	112	–
VS	2922	–	–	–	–	–	–	–	–
VS	2927	*	*	*	*	*	*	*	*
VS	2928	–	–	–	–	–	–	–	–
VS	2939	*	*	*	*	*	*	*	*
VS	2929	*	*	*	*	*	*	*	*

Eight different tissues were collected from the swine challenged with 100 SID_50_ (a) and 10 SID_50_ (b) of FMDV and were analyzed for the viral RNA load by one-step qRT-PCR. The numbers in the columns are the viral RNA copy numbers detected by one-step qRT-PCR.“–” indicates that the viral RNA load in the sample was not detectable. “*” indicates that the sample was not tested.

The TGCS performed better in their ability to clear the FMDV in the tissues than the NS and Non-TGCS. One VS also expressed the viral RNA. The virus level in the tissues exhibited a dramatic decrease in the TGCS, which suggests that the shRNA transgenic swine might have the potential to resist FMDV.

### The antibody response to FMDV

The antibody response against the FMDV structural proteins in the TGCS was later or slower than that in the NS and Non-TGCS. In the 100 SID_50_ FMDV dose challenge study, the FMDV structural protein antibody response of the NS reached the highest average level (> 1024) by 11 d.p.c., and that of the TGCS reached the highest average level by 13 d.p.c. (Supplementary Fig. S6A). The antibody response against the structural protein in the TGCS was slower than in the NS. Moreover, by 7 d.p.c., the level of structural protein antibodies between the TGCS and NS was significantly different (Supplementary Fig. S6A). This finding suggested that the TGCS might have a better anti-FMDV capacity than the NS, and the reason may be that the shRNA expression in TGCS inhibited viral replication in the early period (Supplementary Fig. S6). In the 10 SID_50_ FMDV dose challenge study, the FMDV structural protein antibody response of the TGCS and NS also reached the highest levels by 13 d.p.c. and 11 d.p.c., respectively (Supplementary Fig. S6B). The structural protein antibody response of the TGCS was slightly slower than that of the NS. The levels of antibodies of the two groups detected on these days were not significantly different. Nevertheless, the VS already exhibited a low-level structural protein antibody response at 0 d.p.c. However, one VS swine, #2929, had a very low structural protein antibody level at 0 d and died by 6 d.p.c. (Fig. 3a). Moreover, the 3ABC-antibody (Nonstructural protein antibody) responses of the TGCS and NS or Non-TGCS were not significantly different, and swine #25 and #18 did not have 3ABC-antibody responses (data not shown).

### No significant difference was observed in the number of CD3^+^, CD4^+^, CD8^+^, CD21^+^, or CD172^+^ cells between the TGCS and Non-TGCS or NS

**Figure 4:**
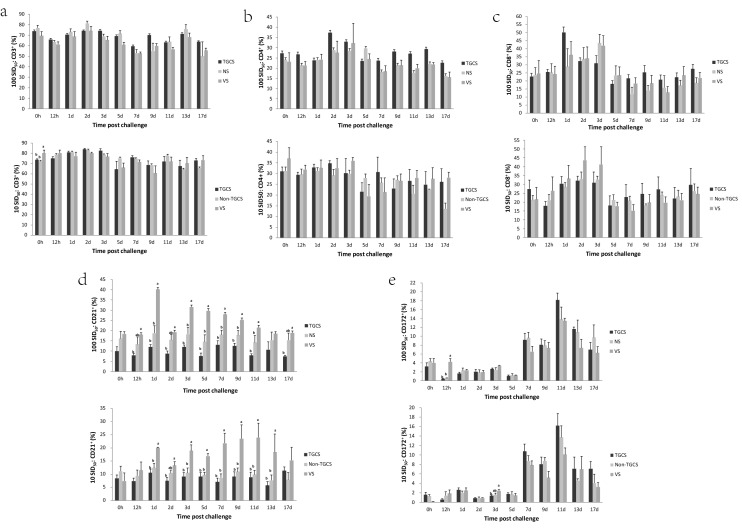
The CD3^+^, CD4^+^, CD8^+^, CD21^+^, and CD172^+^ cells were not significantly different between the TGCS and Non-TGCS or NS groups. The percentage of CD3^+^ (**a**), CD4^+^ (**b**), CD8^+^ (**c**), CD21^+^ (**d**), and CD172^+^ (**e**) cells in the blood samples from 0 h to 17 d was detected by flow cytometry. The error bars represent the standard error. a, b indicates a *P*-value ≤ 0.05, a > b.

The CD3^+^, CD4^+^, CD8^+^, CD21^+^ and CD172^+^ cells were analyzed by flow cytometry. There was a significant difference in the number of CD3^+^ cells in the 10 TCID_50_ challenge study at 0 h between the VS and control groups, but no significant differences were observed between the TGCS and Non-TGCS. There was no significant difference in the number of CD3^+^ cells of the 100 SID_50_ challenge study among groups (Fig. 4a). There was no significant difference in the number of CD4^+^ and CD8^+^ cells among the three groups (Fig. 4b,c). The number of CD21^+^ cells was significantly different when comparing the VS and control groups from 12 h.p.c. to 17 d.p.c. in the 100 SID_50_ challenge study and from 1 d.p.c. to 13 d.p.c. in the 10 SID_50_ challenge study, but there was no significant difference between the TGCS and Non-TGCS or NS at any time point in either study (Fig. 4d). There was also no significant difference in the number of CD172^+^ cells between the TGCS and Non-TGCS or NS in either study. The VS only exhibited a significant difference compared to the control groups at 12 h.p.c. in the 100 SID_50_ study and 3 d.p.c. in the 10 SID_50_ study (Fig. 4e). IFN-γ was detected by one step qRT-PCR in the blood samples from 0 to 5 d.p.c., and our data showed no significant difference among the different groups (Supplementary Fig. S7). These results suggested that the RNAi-based antiviral pathway may mediate the resistance to FMDV in the TGCS.

### Added selective pressure was found in the shRNA target region of the FMDV RNA

The viral RNA was extracted from the viremic blood samples. The 2B, 3D and VP1 genes were amplified by RT-PCR. The RT-PCR products were sequenced, and we identified mutations in the 2B-shRNA and 3D-shRNA target regions of the viral RNA in the TGCS compared to the original viral sequence, but no mutations were observed in the viral RNA in the Non-TGCS and NS. In addition, mutations were identified outside of the shRNA target region in the 2B and 3D genes. There were also mutations in the VP1 gene region (Supplementary Table S2). This finding might indicate that a direct antiviral activity with effective FMDV suppression promoted the added selective pressure of shRNA targeting and confirmed an RNAi mechanism of action.

## Discussion

RNA interference (RNAi) is one of the most exciting new developments in molecular biology. As a natural posttranscriptional gene silencing mechanism, RNA interference has been used in gene function studies, RNAi-based therapies, and antiviral technology^13,50,51^. Here, we designed a vector expressing two shRNAs, which targeted the mRNAs of the 3D polymerase gene and 2B nonstructural protein gene of FMDV and had the potential to interfere with viral replication and packaging. We confirmed the efficacy and specificity of the vector in BHK-21 and IBRS-2 cells. The viral RNA load and virus titer were significantly reduced in the FMDV-infected BHK21-P and IBRS2-P cells compared to the FMDV-infected BHK21-N and IBRS2-N cells. The BHK-21 and IBRS-2 expressing the shRNA had the potential to efficiently inhibit FMDV. Then, we generated shRNA transgenic cloned swine and used five non-transgenic cloned swine as a negative control, which was very rare. These cloned swine shared the same genome background, and the only differences were the presence of the transgene and possibly the epigenome. This similarity made the results more trustworthy. The shRNA transgenic cloned swine displayed resistance to FMDV infection. This phenomenon was also reported with other viruses. The siRNA-mediated anti-SARS study showed that it relieved the symptoms of SCV infection and reduced the SCV viral load and the acute diffuse alveoli damage^33^. A significant decrease in RSV infection was reported in mice, which was associated with the RNAi-mediated knockdown of lung nucleolin^52^. siRNAs inhibit pre- and/or post-integration infection of HIV-1 and virus production by targeting the HIV-1 cellular receptor CD4 and the viral proteins. These studies showed that siRNAs might have the potential to treat HIV-1 and other viral infections^28^.

Genetically modified chickens expressing shRNAs were designed to function as a decoy. All of the non-transgenic chickens and most of the transgenic chickens infected with avian influenza A virus died between 2 and 7 days post-infection. All of the chickens in contact with the non-transgenic chickens that were infected with the virus died and had a high viral RNA load, but most of the chickens that were co-housed with transgenic chickens infected with the virus survived and had a lower viral RNA load. Although the transgenic birds died in the initial experimental challenge, the further transmission was prevented^53^. In our studies, every swine was inoculated successfully with FMDV, one transgenic cloned swine was protected under 100 SID_50_ FMDV challenge, no clinical signs, no viremia, and no viral RNA in the tissues, no non-structural antibody response, and one transgenic cloned swine had recuperated and was healthy after showing lesions for two days in the 10 SID_50_ FMDV challenge study, whereas all of the Non-TGCS and NS developed clinical symptoms, viremia, and viral RNA was determined in the tissues, non-structural antibody was determined., and one Non-TGCS died. The other FMDV-infected TGCS also had a later mean onset of lesions and the longer mean number of days to developing severe lesions. The most important finding was that the viral RNA loads in the blood and tissues of the TGCS were reduced compared to those of the Non-TGCS and NS. Our data indicate that shRNA might help the animal clear the virus.

Our results showed that the vaccine could efficiently protect the swine from developing clinical symptoms, but we found that one vaccinated swine in the VS harbored the viral RNA in two types of tissues. It has been reported that inactivated FMDV vaccines could efficiently prevent clinical disease but do not prevent viral replication after infection. Additionally, FMDV infection can induce persistent infections in approximately 50% of naive and vaccinated animals^54^. A single vaccination was not sufficient to stop swine-to-swine virus transmission^55^. In disease-free countries, there are limitations in using vaccines in the event of an outbreak. Since 1991, the European Union has stopped prophylactic vaccinations^55^. Therefore, there is a need to develop better integrated strategies that fit the specific needs in endemic regions^8^. In this situation, it is important to develop antiviral strategies that are capable of inducing early protection in uninfected, susceptible animals surrounding the disease foci soon after an outbreak has been detected and then block or at least limit the disease spread and virus shedding to potentially reduce the numbers of animals slaughtered^2,7,49^. Here, the shRNA transgenic swine exhibited a delay in clinical symptoms and a reduced viral RNA load, enabling them to generate an antiviral response and induce early protection that can be used in conjunction with vaccines as an integrated strategy to control an FMDV epidemic.

The expression of CD21^+^ cells in VS exhibited a significant difference from that in control groups at many time points. CD21 (CR2, receptor of complement component C3d) expresses on mature B cells and follicular dendritic cells and is part of the B-cell co-receptor complex. This receptor enhances sensitivity to antigens by at least 100-fold. Our results showed that the antibody response against structural proteins in VS was faster than in the control. CD3, CD4, CD8 are the co-receptors on thymocytes and T cells. CD3^+^, CD4^+^, CD8^+^ cell levels in VS had no significant difference from those in control groups. This finding suggested that VS might have a stronger humoral immune response but not the cellular immune response. CD3^+^, CD4^+^, CD8^+^, CD21^+^, and CD172^+^ cell levels were not significantly different between TGCS and Non-TGCS or NS. This finding suggested the TGCS and Non-TGCS or NS might have no difference in adaptive immunity. Mammals have an IFN-based antiviral immune response, which intersects with the RNAi-based antiviral pathway demonstrated in mammals^12,17,18^. IFN-γ was not significantly different among the groups, suggesting that the resistance to FMDV in TGCS may arise from the RNAi-based antiviral pathway.

However, RNAi efficiency still requires improvement. Our results showed that RNAi could not provide complete resistance to FMDV, and the low shRNA expression might be one of the reasons. Here, 25-nt 2B-shRNA expression was tested in transgenic cell lines and swine by real-time qRT-PCR, but it could not be detected by Northern blotting, possibly because of the expression level was below the detection threshold for this assay. In Drosophila and mammals, 21- to 25-nt siRNAs derive primarily from the cleavage of longer double-stranded RNA (dsRNA) molecules by DICER nuclease and trigger RNAi, which guide the cleavage of homologous RNA molecules^56,57^. In our study, the 56-nt 3D-shRNA was cleaved into different siRNA types; thus, it was difficult to detect this shRNA either by Northern blotting or real-time qRT-PCR^53^. However, it may be detectable by deep sequencing. Although we did not detect the expression of 3D, viral RNA sequencing revealed a high mutant base ratio in both the 2B-shRNA and 3D-shRNA target regions of viral RNA in TGCS after challenge, but no mutation was found in this regions of viral RNA in Non-TGCS or NS. In addition, a lower mutant base ratio was found out of the shRNA target region in 2B and 3D genes. This finding indicated that shRNA targeting could cause selective pressure on the viral RNA and trigger selective mutations to improve survival. Furthermore, it also indicated 2B-shRNA and 3D-shRNA were successfully expressed and exhibited a direct antiviral activity with effective FMDV suppression, confirming an RNAi-mediated mechanism of action. RNA viruses typically have a high mutation rate; thus, it has been suggested that multiple shRNAs be used to overcome this problem^58^.

In fungi, plants, and *C. elegans*, two different siRNA populations—“Primary siRNAs” and “Secondary siRNAs”—have been proposed to participate in RNAi. “Primary siRNA” derives from DICER nuclease-mediated cleavage. Along with this “primary” siRNA response, amplification of the RNA requires RNA-directed RNA polymerase (RdRP). “Secondary siRNAs” constitute the vast majority of siRNA populations^56^. Viral dsRNA is cleaved into siRNAs, and the secondary siRNAs can be produced through amplification pathways; these siRNAs are able to spread from cell to cell to inhibit the spread of the virus^59–61^. However, RdRP does not appear to be present in mammals^62^. Future studies should investigate the production of transgenic cloned swine that express RdRP, which might be a way to amplify the expression of siRNAs in swine and generate a better antiviral response.

Our data revealed no obvious difference between these two challenge studies, which may be because the doses we used were both sufficiently to be effective in swine (1 SID_50_ is the dose to infect 50% of the swine, and we used 10 SID_50_ and 100 SID_50_). Moreover, all of the transgenic cloned swine showed different levels of resistance to the virus, although their genomes were identical, as were those of the non-transgenic cloned swine. In addition, we did not observe a significant difference in the siRNA expression among the transgenic swine, perhaps as a result of epigenetic differences.

Our results showed that RNAi promoted the anti-FMDV response in both cells and the transgenic cloned swine. Here, we used five non-transgenic cloned swine as a negative control, which shared the same genomic background as the transgenic cloned swine. The transgenes confer the swine with resistance to FMDV via the RNAi pathway, which could be a great help during a sudden FMDV outbreak, particularly in countries that do not use vaccines. Transgenic swine can also be treated with vaccines as an integrated strategy to control an FMDV epidemic. And if we can improve the expression of the siRNAs via transgene-induced amplification, identify better targets, such as the viral genes that suppress the host RNAi pathway, or express multiple shRNAs, then we could greatly improve the anti-FMDV efficiency. Our work provides a foundation for anti-infectious disease breeding studies in swine, and the shRNA transgenic technology might be a valuable approach to solving the major problem of FMDV.

## Materials and methods

### Ethics approval and consent to participate

All of the experiments performed in compliance with the Animal Research: Reporting in vivo Experiments guidelines, and were approved by the Animal Ethics Committee of China Agricultural University (License No. SKLAB-2010-04-02, Beijing, China) and Gansu Animal Experiments Inspectorate and Ethical Review Committee (License no. SYXK (GAN) 2010-003, Lanzhou, Gansu, China) for the use of animals in scientific research. And all of the experiments followed the standard protocol described by the OIE for the virus used. All the animals were euthanatized with a barbituric overdose. The study was carried out in compliance with the ARRIVE guidelines.

### Construction of an shRNA-expressing vector

The shRNA-expressing plasmid PB-EN3D2B was kindly provided by Prof. Zhaoxin Zheng^63^. The two repeat shRNA coding sequences, EGFP and Neo-R expressing sequences were digested from the piggyBac vector (PB-EN3D2B) and then inserted into the pMD19-T vector (Takara, Japan). The new vector was named pEN3D2B. The mouse U6 promoter (mU6) and human H1 promoter (hH1) were used to initiate the expression of 3D-specific shRNA (3D-shRNA) and the 2B-specific shRNA (2B-shRNA), respectively, and five T residues were used as the terminal signal. The mPGK promoter was used to initiate EGFP expression, which ended with an SV40-polyA tail. In the same way, Neo-R was initiated by the SV40 promoter and ended with an HSV-TK-polyA tail (Supplementary Fig. S1A). The shRNA expression vector was named p3DEN2B and targets either nonstructural protein 2B or polymerase 3D of FMDV. The control vector, pEN, which only expresses EGFP and Neo-R, but not the shRNA, was also constructed (Supplementary Fig. S1B).

The sequence of the 25-nt oligonucleotide encoding the FMDV 2B-shRNA was 5′-CCAGATGCAGGAGGATATGTCAACA-3′; the sequence of the 56-nt oligonucleotide encoding the FMDV 3D-shRNA was 5′-GAGGCCATCCTCTCCTTTGCACGCCGTGGGACCATACAGGAGAAGTTGATCTCCGT-3′.

### Preparation and identification of the transgenic cell lines

Baby hamster kidney cells (BHK-21, China Center For Type Culture Collection) and swine kidney cells (IBRS-2, China Center For Type Culture Collection) were used in the antiviral assays. All of the cell lines were cultured in HyClone DMEM with 10% HyClone fetal bovine serum in a 37 °C incubator with 5% CO_2_.

The vectors p3DEN2B and pEN were linearized by Pvu I. The fragment containing the shRNA, EGFP, and NEO expression sequences from p3DEN2B and the EGFP and NEO sequences from the pEN expressive sequence were purified and concentrated; 1 μg/μL of the concentrated linearization plasmid was transfected into the BHK-21 and IBRS-2 cells, respectively, using the Amaxa Nucleofector™ II Device. The transfected cells were revived in selected culture medium with G418; after one week, we harvested the NEO-positive cells. The cells were digested with trypsin and resuspended in the culture medium without fetal bovine serum. Then, the cells with the strongest GFP expression were sorted by the Beckman MoFlo™ XDP Flow Cytometer. The GFP-expressing cells were revived in culture medium with 10% fetal bovine serum; we detected the GFP expression using a fluorescence microscope and ascertained that the cells were all transgene positive. All four types of transgenic cells were frozen in a freezing medium (DMSO:fetal bovine serum:DMEM = 1:3:6) and stored in liquid nitrogen.

### In vitro viral challenge assay using BHK-21 and IBRS-2 cells

The FMDV strain Guangdong/CHA/86 [GenBank accession AJ131468], Serotype O, was isolated in 1986 in Guangdong, China (provided by Lanzhou Veterinarian Research Institute). This strain was used in the challenge experiments.

We used cultured the BHK-21 cells to grow and titrate the FMDV. The 50% tissue culture infective doses (TCID_50_) were calculated using the Reed-Muench formula. Viral suspensions titrated at 10^7^ TCID_50_/mL were used in the experiments. The four transgenic cell lines—BHK-P, BHK21-N, IBRS2-P and IBRS2-N—were plated in 96-well plates separately. A few days later, the cells were approximately 95% confluent. A dose of 100 TCID_50_ of FMDV per 0.1 mL was added to each well. The infection was allowed to proceed without removing the FMDV. The virus titers (TCID_50_) were determined three times using BHK-21 cells.

The four cell lines BHK-P, BHK21-N, IBRS2-P, and IN were plated in twelve-well plates. After reaching 95% confluence, 10 TCID_50_ or 100 TCID_50_ of FMDV per mL was added to each well, except for the 0 h control plates. After one h of incubation, the virus was removed and then 1 mL of new DMEM with 10% fetal bovine serum was added. At the designated time (12, 24, and 48 h), one plate of each cell line was removed from the incubator. The cells were examined for cytopathic effects (CPE) and GFP expression using a fluorescence microscope. Then, the plates were sealed and frozen at − 80 °C. After freeze–thaw cycles, the FMDV was released from the cells. We then added 1 mL of TRIzol reagent (Invitrogen), mixed the samples, and transferred them to 2 mL tubes. The total RNA was extracted from the infected cell samples and quantitated by One-step qRT-PCR^64^ using a Stratagene Mx3005P QPCR machine (Agilent Technologies). The TaqMan^®^ probe (SAmulti2-P-IR-292-269R) and primers (SA-IR-219-246F, SA-IR-315-293R) were used to target the conserved sequences of the FMDV genome, which is an internal ribosomal entry site of the 5′-untranslated region. Twenty microliters of the qRT-PCR master mix and 5 μL of the RNAs were added to a 96-well optical reaction plate (Stratagene, La Jolla, CA). The qRT-PCR master mix was pre-prepared and included 0.5 μL High Fidelity Enzyme Mix (SuperScript III/Platinum Taq One-Step qRT-PCR Kit; Invitrogen), 1.5 μL nuclease-free H2O (Promega, Madison, WI), 1.5 μL probe (5 pmol/μL), 2.0 μL of each primer (both at 10 pmol/μL), and 12.5 μL 2 × reaction mix (SuperScript III/Platinum Taq One-Step qRT-PCR Kit; Invitrogen, Carlsbad, USA). Briefly, the reaction was performed using the following thermal profile: 30 min at 60 °C, one cycle; 10 min at 95 °C, one cycle; 15 s at 95 °C and 1.06 min at 60 °C, 50 cycles. Each reaction was run in triplicate. The results were analyzed by the Stratagene^®^ MxPro™ QPCR software 3.0, and a CT value was assigned to each reaction^65^. The samples with a CT value of 36 or less were considered positive. Quantification was relative to a standard curve obtained with known amounts of the FMDV RNA. The standard formula is y =  − 3.416log(x) + 42.85, and the correlation coefficient is 0.999; where y is the mean CT and x is the mean copy number of the FMDV RNA.

### Quantification of shRNA expression using real-time PCR

Small RNAs (20–200 nt) were purified from the tissues and cells using the miRcute miRNA Isolation kit (TIANGEN) according to the manufacturer’s recommendations. Real-time quantification of the siRNA was performed by adding polyA and RT-PCR. First, PolyA was added to the 3′ end of the siRNA using *E. coli* polyA polymerase, and then the PolyA reaction solution was used for the first-strand cDNA synthesis using the miRcute miRNA First-strand cDNA Synthesis kit (TIANGEN) protocol. Real-time PCR was performed using an miRcute miRNA qPCR Detection kit (TIANGEN) on an MX3000p system from Stratagene, and the data were analyzed using the MX3000p software provided by the manufacturer. Each reaction was run in triplicate. We used U6 as the internal DNA control, the primers was designed for 2B siRNA and U6. The primers used for the experiment were as follows: 2BshRNA_F: 5′-GTCACCAGATGCAGGAGGATATG-3′; U6_F: 5′-CGCAAATTCGTGAAGCGTTC-3′. All of the reverse primers were provided with the kit.

### Generation and identification of the shRNA transgenic cloned swine

The linearized p3DEN2B construct (the same with the constuct used in cell lines) was transfected into the swine fibroblast cell line using the Amaxa Nucleofector™ II Device. The transfected fibroblasts were cultured and passaged in selected culture medium with G418 (400 µg/mL, Promega) for fourteen days. The antibiotic-resistant colonies were selected and identified by PCR to confirm that the p3DEN2B vector had been inserted. The fibroblasts containing the vector were used as the nuclear donors in the somatic nuclear transfer (SCNT) procedure. SCNT was performed as previously described^66^ (Fig. 2).

### Viral challenge of the swine and clinical analysis

Ten transgenic cloned swine and five non-transgenic cloned swine were transported to the National P3 Lab at the Lanzhou Veterinarian Research Institute for the challenge experiments. The cloned swine were all 3-month-old Landrace male swine weighing ca. 40–50 kg. The wild-type swine were used to perform a viral challenge as the normal control and vaccine-inoculated control. None of the swine had previous FMDV contact, as confirmed by the absence of detectable anti-FMDV antibodies (use LPBE as below) or antibodies against other viruses, such as porcine reproductive and respiratory syndrome virus (PRRSV), classical swine fever virus (CSFV), and porcine circovirus 2 (PCV2), in their serum.

The virus CHA/86 used to challenge was titrated in wild-type swine. The dose of FMDV used was determined by means of four tenfold serial dilutions of the virus (i.e., 10^-4^, 10^-5^, 10^-6^, and 10^-7^) in phosphate-buffered saline (PBS). Sixteen swine were divided into four groups (four swine/each group). Each animal was inoculated in the neck region by intramuscular injection of 3 mL of the serially diluted virus. All of the animals were monitored twice per day for the major clinical symptoms of FMD, namely, mouth and foot lesions. The 50% swine infective dose (SID_50_) was estimated as 6.0/3 mL according to the Reed-Muench method (Reed et al. 1938).

All of the animals were housed in disease-security isolation facilities in the P3 Lab. Twelve control swine were inoculated with 2 mL of vaccine (Swine Foot and Mouth Disease Type O vaccine, Inactivated II, China Agricultural Vet. Bio. Science and Technology Co., Ltd.) by intramuscular injection in the neck area three weeks before the viral challenge. Ten swine were randomly chosen as the vaccinated controls. The swine without vaccination was used as the normal swine controls. Ten transgenic cloned swine, 10 wild-type swine, and 10 vaccine immunized swine were equally divided into two challenge studies (100 SID_50_ and 10 SID_50_). All animals were challenged by intramuscular injection with 3 mL of 100 SID_50_ or 100 SID_50_ of CHA/86.

After the challenge, the swine that developed the disease were moved to another room to avoid overexposure to the challenge virus, and then the observation proceeded. The swine were fed with the standard diet. The room temperature and humidity were set to the same level, and each room had an independent vented air filter system to prevent contamination. The animals were monitored twice daily for the clinical symptoms of FMD, including an increase in rectal temperature and mouth and foot lesions. The clinical lesion scores were determined at various time points following a challenge by the method established by Pacheco^67^. The clinical lesion scores for the swine were based on the sites containing the FMD lesions (vesicular lesions, erosion of epithelium, and blanching of the coronary band). One point was awarded for affected digit on each foot or the three sites (tongue, snout, and lower lip) bearing one or more vesicles within ≤ 1.0 cm in diameter. Two points were awarded for affected digit on each foot or any of the three sites (tongue, snout, and lower lip) bearing one or more vesicles within ≤ 2.0 cm in diameter. Three points were awarded for affected digit on any of the three sites (tongue, snout, and lower lip) bearing one or more vesicles within > 2.0 cm in diameter. Six points were awarded for affected digit on each foot bearing one or more vesicles within > 2.0 cm in diameter. The maximum lesion score for the swine was 27. The scores for each swine were recorded daily until the vesicles at all sites had started to heal. The observations were terminated on day 12 post-challenge in the swine experiments.

### Quantification of the viral RNA load in the swine serum and tissue samples

Serum samples were collected at every selected time point until 17 days post-challenge (d.p.c.). Tissue samples (heart, liver, spleen, lung, kidney, submaxillary lymph nodes, mesenteric lymph nodes, tonsil) were collected after necropsy at 17 d.p.c. Automated viral RNA purification was performed using 100 µL of the serum samples or 100 mg of the tissues, according to the QIAxtractor (Qiagen) protocol, and then the viral RNA loads were analyzed by one-step real-time quantitative RT-PCR (one-step qRT-PCR) according to the procedure described above.

### Detection of the structural protein (SP) and nonstructural protein (NSP) antibodies

The serum samples collected at 0–17 d.p.c. were used to examine the antibodies against the FMDV structural protein (SP) by Liquid-Phase Blocking ELISA (LPBE). LPBE was performed according to the standard method of the OIE to determine the antibody titers of the candidate swine before and after the challenge. The LPBE kits were from Lanzhou Veterinary Research Institute. The samples were considered positive if the percentage inhibition was 50 or greater. Swine with a potency lower than 4 were used as the normal control in the viral challenge assay.

The nonstructural protein (NSP) 3ABC Indirect Enzyme-linked Immunosorbent Assay (I-ELISA) was previously established^68^ and uses purified His-tagged 3ABC fusion protein as the antigen to detect the antibody response to FMDV NSP 3ABC in different animal species. The NSP 3ABC-I-ELISA kits were from Lanzhou Veterinary Research Institute. The OD value of the positive control (ODpos) and the samples (ODsample) were corrected by subtracting the OD value of the negative control (ODneg). The sample value was calculated as a ratio using the formula value = (ODsample − ODneg)/(ODpos − ODneg); sample value > 0.3, positive; sample value ranging from 0.2 to 0.3, suspicious; sample value < 0.2, negative. The serum samples classified as suspicious were repeatedly confirmed by the same method. If the result was repeated, the sample was judged to be positive.

### Cell staining and flow cytometry analysis

Flow cytometry was performed as previously described to analyze the cells expressing the surface molecules CD3, CD4, CD8, CD21, and CD172^69^. The antibodies used were a mouse anti-swine CD3-PE/CyTM5 tandem conjugate, mouse anti-swine CD8-fluorescein (FITC) conjugate and mouse anti-swine CD4-R-phycoerythrin (R-PE) conjugate (all from SouthernBiotech, USA). A mouse anti-swine CD21-R-phycoerythrin (R-PE) conjugate (SouthernBiotech, USA) was used for B cell staining. A mouse anti-swine CD172-fluorescein (FITC) (AbD Serotec, UK) antibody was also used^69^. According to the protocol recommended by the manufacturer, the lymphocytes from blood samples were stained with the surface antibodies listed above, and the stained cells were analyzed by flow cytometry using a FACScan flow cytometer and CellQuest software (BD Biosciences, San José, CA, USA).

### Quantification of the IFN-γ mRNA

The IFN-mRNA levels in the blood samples from 0 h to 5 d were tested by one-step qRT-PCR^70^. IFN-mRNA expression was determined using the RNeasy Plus Mini Kit for RNA extraction (Qiagen, Valencia, CA) and TaqMan One-Step qRT-PCR kit for RT-PCR (Applied Biosystems, Carlsbad, CA). The levels of gene expression were normalized to β-actin for each sample. The fold increase in gene expression was determined using the ^△△^CT method. The samples were run in triplicate, and the results are presented as the linear fold changes in gene expression.

### Synthesis, amplification, and cloning of the FMDV cDNA

FMDV RNA was extracted from the viremic blood samples of the TGCS, Non-TGCS and NS groups. The cDNAs were synthesized from the viral RNA. The following primers were used to amplify the FMDV cDNA: 2B-F1:5′-TCTTCTTCTCCGACGTCAGGTC-3′ (5–26); 2B-R1:5′-CTTCACTACAAAGGGGCTGTCC-3′ (294–315); 3D-F1:5′-GGTCAAACCATTACTCCAGCCG-3′ (1081–1102); and 3D-R1:5′-CGTTCACCCAACGCAGGTAAAG-3′ (1372–1393). The RT-PCR (Takara) products of the VP1 gene, 2B gene and 3D gene were 639 bp, 267 bp and 269 bp, respectively. The amplified products were digested and ligated to T-vector pMD20 (Takara). The resulting recombinant plasmids were sequenced using a pMD20-T F13 primer (5′-TTCGAGCTCGGTACCCGGGGATCCGATT-3′) and pMD20-T F47 primer (5′-AATCCATATGACTAGTAGATCCTCTAG-3′). Approximately 40 clones from each sample (TGCS, Non-TGCS and NS groups) were sequenced to detect the variants. All cloned FMDV sequences were aligned against the sequence of the Guangdong/CHA/86 [GenBank accession AJ131468] using MegAlign.

### Statistical analysis

SPSS Statistics 17.0 software was used for all of the statistical analyses.

## Conclusions

FMDV is one of the most important animal pathogens in the world. FMDV naturally infects swine, cattle, and other cloven-hoofed animals. FMD is not adequately controlled by vaccination. An alternative strategy is to develop swine that are genetically resistant to infection. Twenty-three transgenic cloned swine (TGCS) and nine Non-transgenic cloned swine (Non-TGCS) were produced by somatic cell nuclear transfer (SCNT). In the FMDV challenge study, one TGCS was completely protected, no clinical signs, no viremia and no viral RNA in the tissues, no non-structural antibody response, whereas all of the normal control swine (NS) developed typical clinical signs, viremia, and viral RNA was determined in the tissues, the non-structural antibody was determined. These results indicated that the TGCS displayed resistance to the FMDV infection. Our work provides a foundation for a breeding approach to preventing infectious disease in swine.

## Supplementary Information

Supplementary Information.

## Data Availability

The datasets during and/or analysed during the current study available from the corresponding author on reasonable request.
