# Supplementary material for: shRNA transgenic swine display resistance to infection with the foot-and-mouth disease virus
Source: Sci Rep. 2021 Aug 12;11:16377. doi: 10.1038/s41598-021-95853-3 (PMC8361160; doi:10.1038/s41598-021-95853-3)
Supplement: Supplementary file 1 — Supplementary Information. [file 41598_2021_95853_MOESM1_ESM.docx]

**shRNA Transgenic Swine Display Resistance to Infection with the Foot-and-Mouth Disease Virus**

Wenping Hu^1,3#^, Haixue Zheng^2#^, Qiuyan Li^1,4^, Yuhang Wang^1^, Xiangtao Liu^2^, Xiaoxiang Hu^1^ , Wenjie Liu^1^, Shen Liu^1^, Zhisheng Chen^1^, Wenhai Feng^1^ , Xuepeng Cai^2*^, Ning Li^1*^

^1^ State Key Laboratory of AgroBiotechnology, China Agricultural University, Beijing, China; [liqiuyan-yuan@163.com](mailto:liqiuyan-yuan@163.com) (Q.L.); [yuhang-077@163.com](mailto:yuhang-077@163.com) (Y.W.); [yaofengzhao@hotmail.com](mailto:yaofengzhao@hotmail.com) (Y.Z.); [xiaoxiang.hu@gmail.com](mailto:xiaoxiang.hu@gmail.com) (X.H.); [liuwenjie213@126.com](mailto:liuwenjie213@126.com) (W.L.); [liushen163@163.com](mailto:liushen163@163.com) (S.L.); [cauchen.zs@gmail.com](mailto:cauchen.zs@gmail.com) (Z.C.); [07003@cau.edu.cn](mailto:07003@cau.edu.cn) (W.F.); [ninglcau@sina.com](mailto:ninglcau@sina.com) (N.L.)

^2^ State Key Laboratory of Veterinary Etiological Biology, National Foot and Mouth Diseases Reference Laboratory, Lanzhou Veterinarian Research Institute, Chinese Academy of Agricultural Sciences, Lanzhou, Gansu Province, China; [haixuezheng@163.com](mailto:haixuezheng@163.com) (H.Z.); [liuxiangtao@caas.cn](mailto:liuxiangtao@caas.cn) (X.L.); [caixp@vip.163.com](mailto:caixp@vip.163.com) (X.C.)

^3^ Key Laboratory of Animal Genetics and Breeding and Reproduction of Ministry of Agriculture and Rural Affairs, Institute of Animal Sciences, Chinese Academy of Agricultural Sciences, Beijing 100193, China; [huwenping@caas.cn](mailto:huwenping@caas.cn) (W.H.)

^4^ Beijing Genprotein Biotechnology Company, Beijing, China; [liqiuyan-yuan@163.com](mailto:liqiuyan-yuan@163.com) (Q.L.)

***** Correspondence: [caixp@vip.163.com](mailto:caixp@vip.163.com)(X.C.); [ninglcau@sina.com](mailto:ninglcau@sina.com) (N.L.)

**^#^** These authors contributed equally to this work.

**
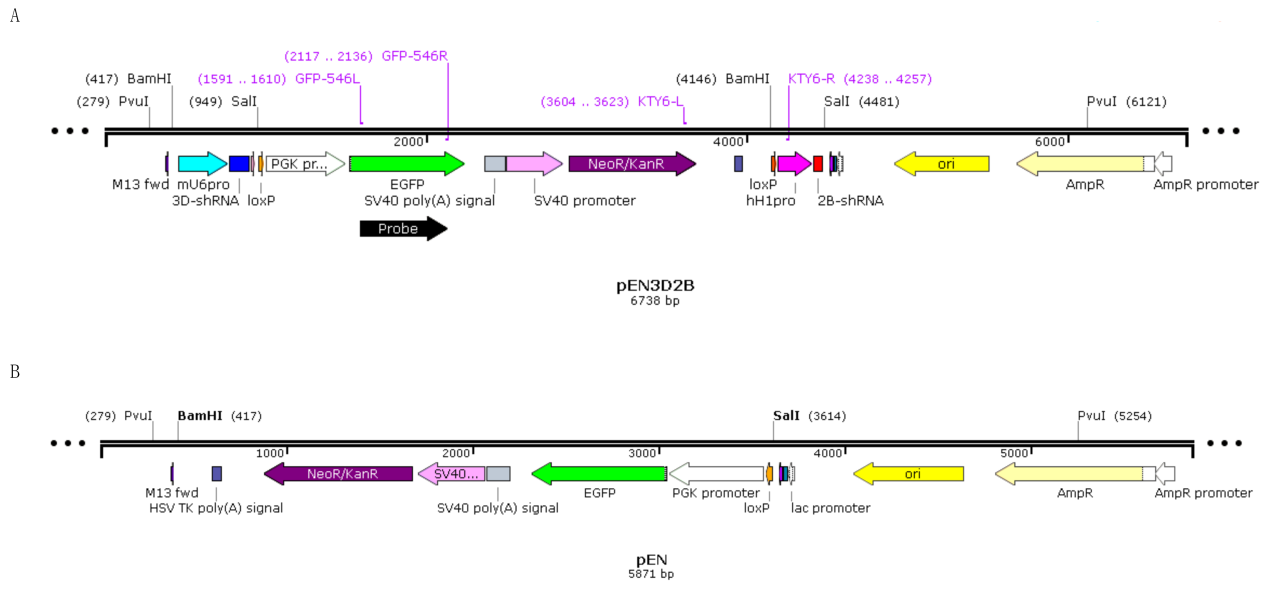
**

**Figure S1. The shRNA expression vector sequence map used for transgenic modification.** (A) pMD19-3D-EGFP-NEO-2B (p3DEN2B): shRNA-expressing vector, expressing the 3D-shRNA, EGFP, Neo-R, and 2B-shRNA. The backbone vector is pMD19 with two loxP sites flanking the reporter gene. The black array shows the probe targeting EGFP that was used in the Southern blot assay; primers KTY6-L and KTY6-R were used in the PCR assay. (B) pMD19-EGFP-NEO (pEN): Control vector, has the same backbone vector as p3DEN2B but does not have the shRNA-expressing cassette and only expresses EGFP and Neo-R.


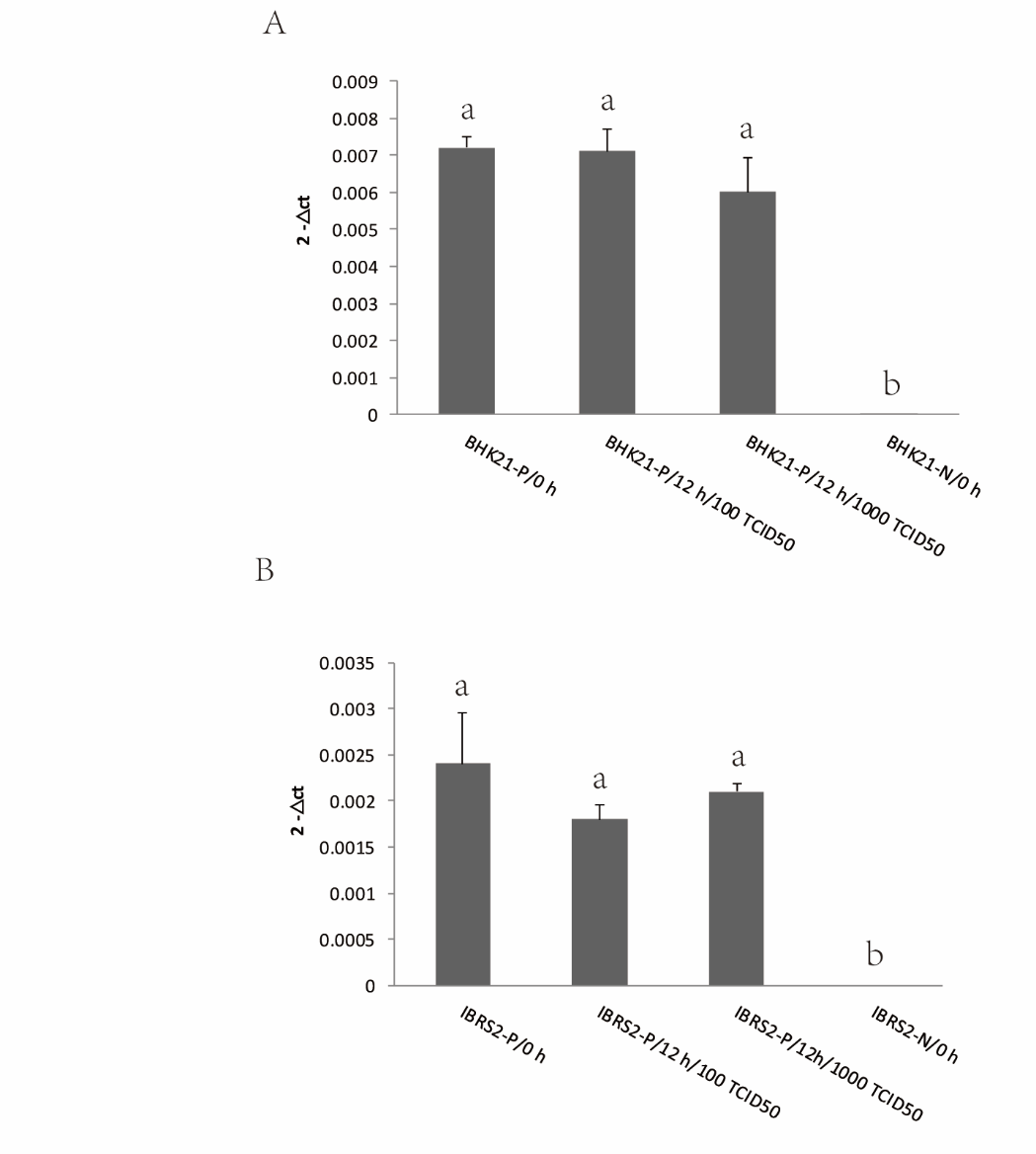


**Figure S2.** **Analysis of 2B-siRNA expression in the transgenic cell lines.** Total small RNAs were isolated from the BHK-21 and IBRS-2 cell line samples. Real-time PCR was used to determine the 2B-siRNA expression. The U6 RNA was used as a reference gene, and the relative expression of the siRNA is expressed as a ratio to U6. (A) 2B-siRNA expression in different BHK-21 cell lines. BHK21-P / 0 h: BHK21-P sample at 0 h before infection; BHK21-P / 12 h / 10 TCID_50_: BHK21-P sample at 12 h following infection with 10 TCID_50_ of FMDV; BHK21-P / 12 h / 100 TCID_50_: BHK21-P sample at 12 h following infection with 100TCID_50_ of FMDV; BHK21-N / 0 h: BHK21-N sample at 0 h before infection. (B) 2B-siRNA expression in the different IBRS-2 cell lines. IBRS2-P / 0 h: IBRS2-P sample at 0 h before infection; IBRS2-P / 12 h/ 10 TCID_50_: IBRS2-P sample at 12 h following infection with 10 TCID_50_ of FMDV; IBRS2-P / 12 h / 100 TCID_50_: IBRS2-P sample at 12 h following infection with 100 TCID_50_ of FMDV; IBRS2-N / 0 h: IBRS2-N sample at 0 h before infection. The error bars represent the standard error. a, b indicates a P-value ≤ 0.05. a > b. The samples are labeled as in Fig 1.


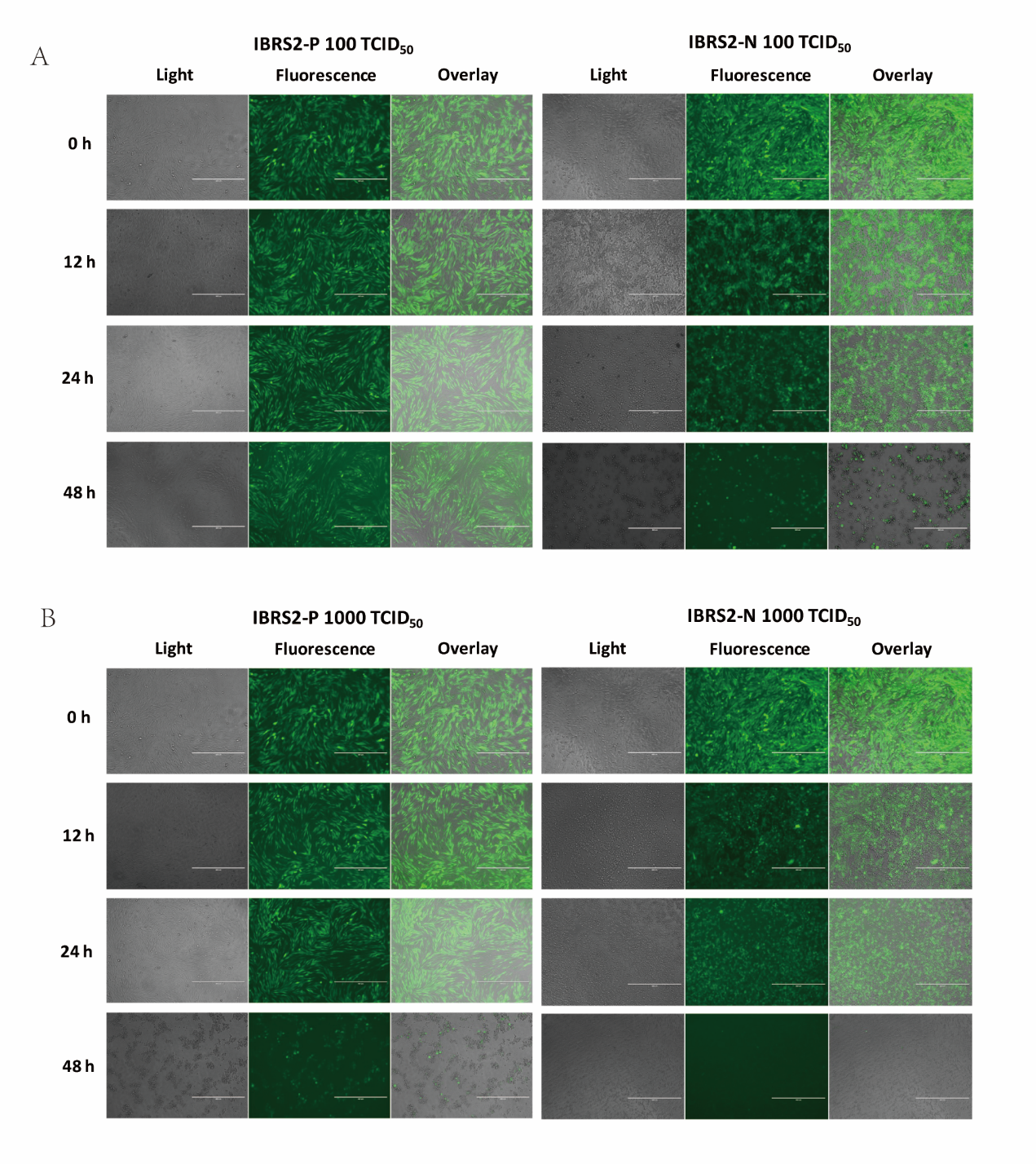


**Figure S3. shRNA expression in the BHK-21 and IBRS-2 cells conferred resistance to FMDV infection.** The IBRS2-P and IBRS2-N cell lines were challenged with 100 or 1000 TCID_50_ of FMDV. These two cell lines can express GFP. The cytopathic effect (CPE) was observed at 0, 12, 24, and 48 h.p.i. under a microscope able to show the image under normal white light (Light) and green fluorescence (Fluorescence) as well as the overlay of these two images (Overlay). (A) 100 TCID_50_ FMDV-challenged IBRS-2 cell lines. (B) 1000 TCID_50_ FMDV-challenged IBRS-2 cell lines.

**
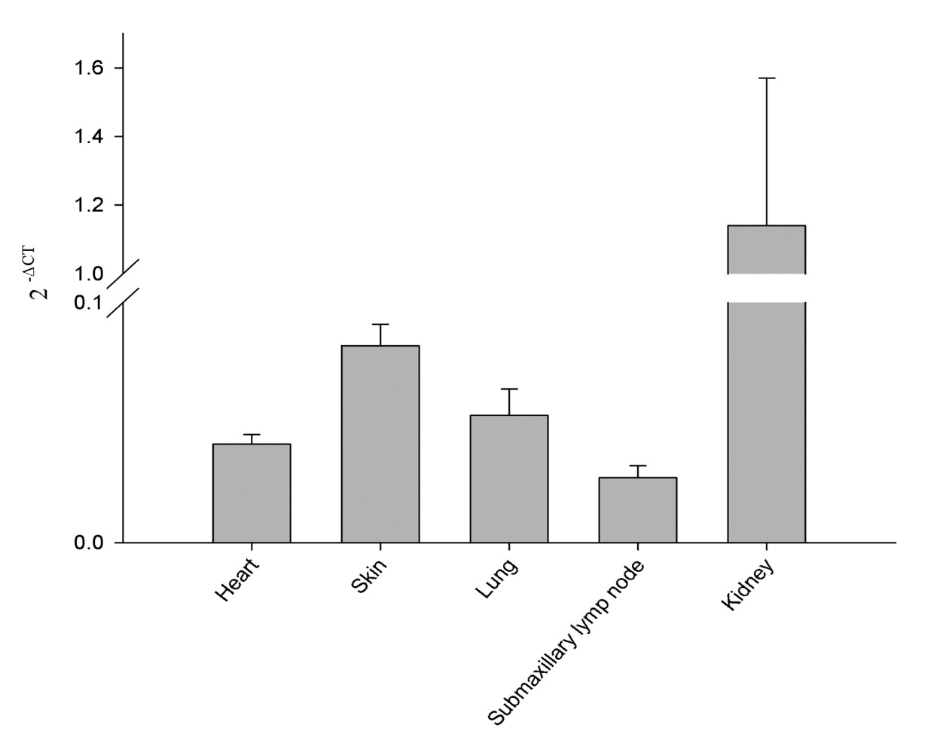
A**


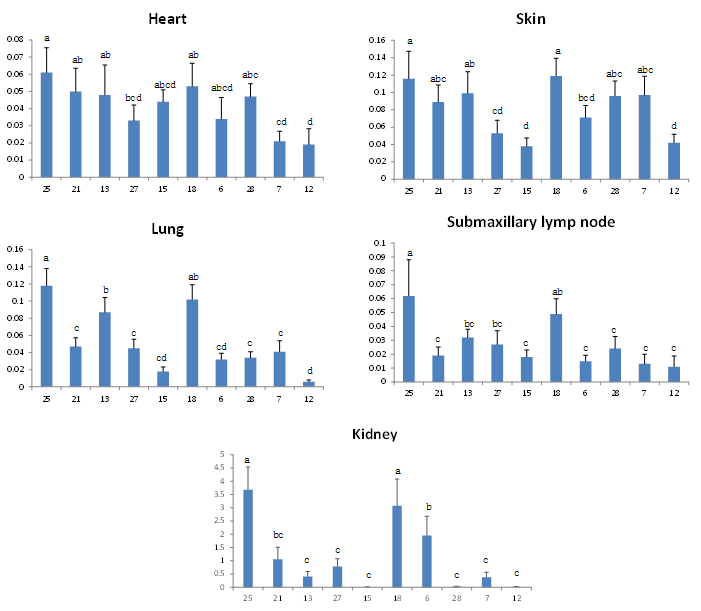
**B**

**Figure S4. siRNA expression analysis of the cloned swine by qRT-PCR.** (A) The average siRNA expression level of the cloned swine. The data are presented as the means ± SE from 10 TGCS swine.The error bars represent the standard error. (B) Expression level of siRNA results in tissues of 10 TGCS. The abscissa number represented different TGCS number, and the ordinate was the expression level of 2B siRNA. RNA was isolated from the TGCS tissue samples (heart, skin, lung, submaxillary lymph node, and kidney), and 2B siRNA expression was quantified by real-time RT-PCR; the U6 RNA was used as an internal control. The error bars represent the standard deviation.

**
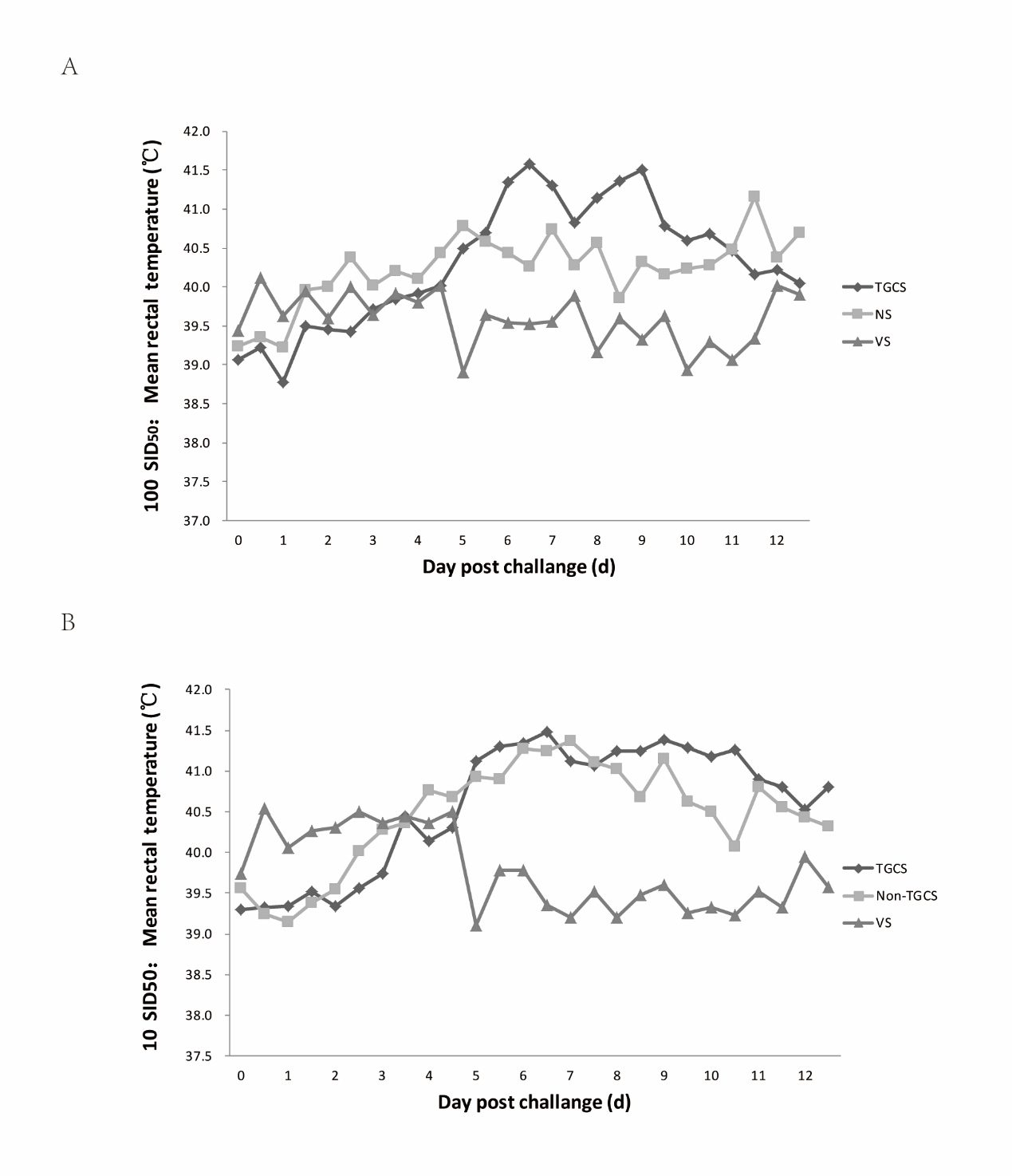
**

**Figure S5. Swine mean rectal temperature in the 10 SID_50_ (A) and 100 SID_50_ (B) FMDV dose challenge study.** The normal rectal temperature is approximately 38.7°C - 40°C.

**
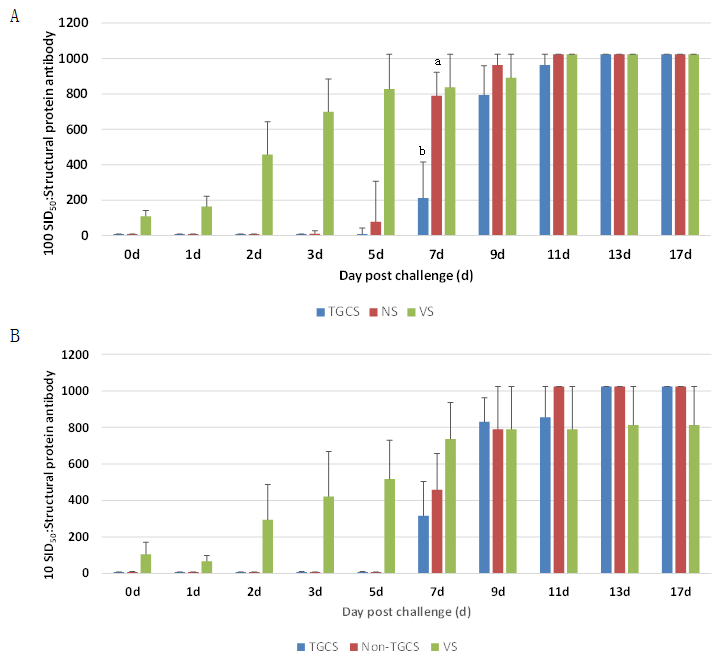
**

**Figure S6. Structural protein antibody titer in the blood before and after challenge with 100 SID_50_ (A) or 10 SID_50_ (B) of FMDV.** The short bar represents the standard error.Because of the VS had a low level structure antibody expression in the beginning of challenge, and the structure antibody started raising up very quick after challenge, so here we did not compare the other groups with VS. We just compared TGCS with NS, or TGCS with Non-TGCS. a, b Means in the same day post challenge with different superscripts differ (*P* < 0.05) between TGCS and NS or Non-TGCS, a > b.


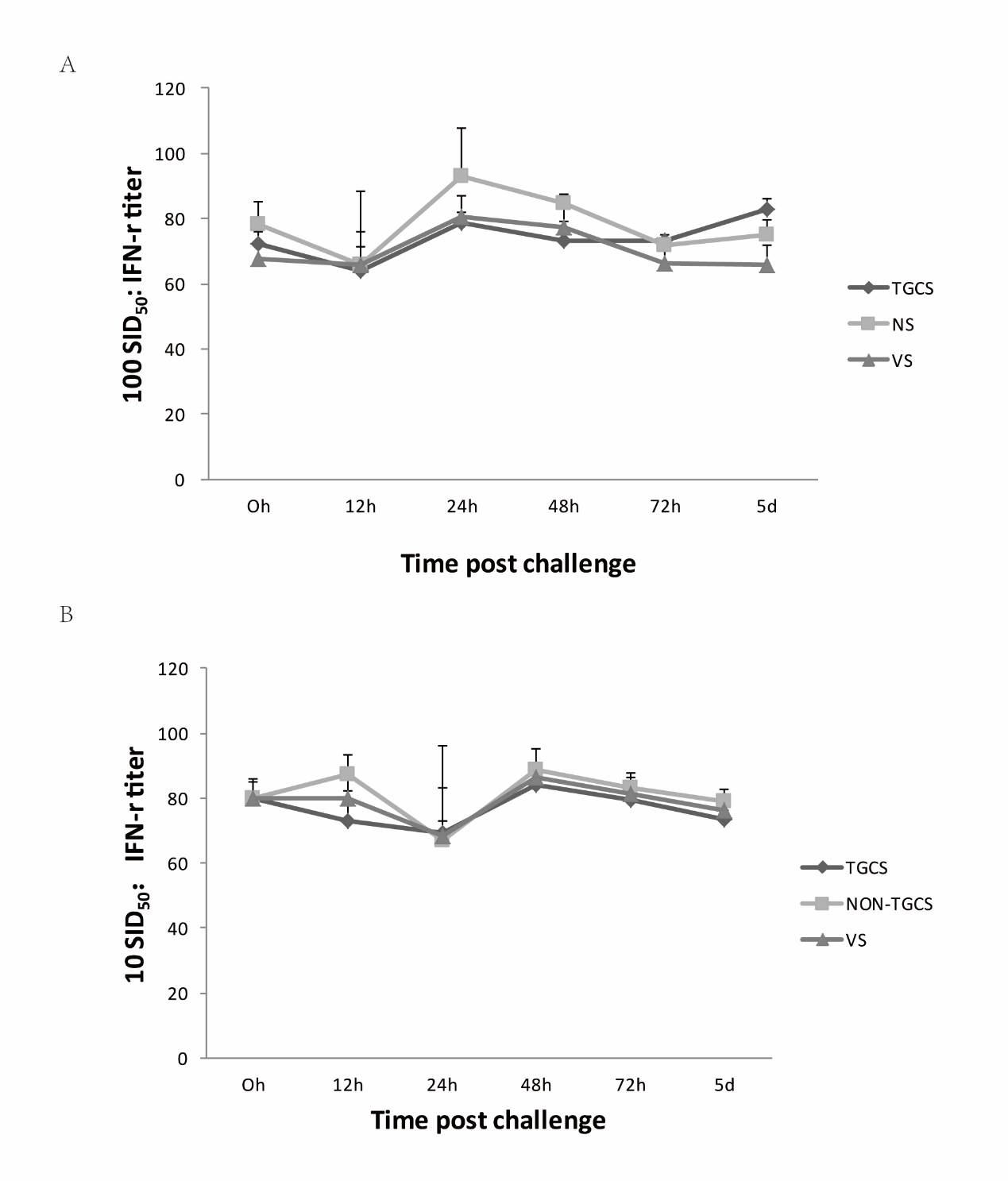


**Figure S7. The IFN-γ response in swine was not significantly different.** The IFN-γ titer in the blood samples from 0 d to 5 d was tested by one-step qRT-PCR. The error bars represent the standard error.

**Table S1. Viremia in swine at various time points after challenge with 100 SID_50_ (A) and 10 SID_50_ (B) of FMDV.**

**A**

| Group | Animal number | Viremia（100 SID_50_ FMDV challenge) | | | | | | | | | | | |
| --- | --- | --- | --- | --- | --- | --- | --- | --- | --- | --- | --- | --- | --- |
|  |  | 0 d | 0.5 d | 1 d | 2 d | 3 d | 5 d | 7 d | 9 d | 11 d | 13 d | 15 d | 17 d |
| TGCS | 25 | - | - | - | - | - | - | - | - | - | - | - | - |
| TGCS | 21 | - | - | - | - | - | - | 71871.6 | - | - | - | - | - |
| TGCS | 13 | - | - | - | - | - | - | 2278.9 | - | - | - | - | - |
| TGCS | 27 | - | - | - | - | - | - | 3324 | - | - | - | - | - |
| TGCS | 15 | - | - | - | 218.3 | 3438 | 59 | - | - | - | - | - | - |
| NS | 2938 | - | - | - | - |  | 5699.7 | 115.8 | 92.6 | - | - | - | - |
| NS | 2978 | - | - | - | 373.8 | 1951.6 | 159.6 | - | - | - | - | - | - |
| NS | 2968 | - | - | - | 58.2 | 1302.4 | 1092.4 | 270.8 | 47.3 | - | - | - | - |
| NS | 3276 | - | - | 437 | 65399.4 | 26.3 | - | - | - | - | - | - | - |
| NS | 2967 | - | - |  | 911.2 | 691.2 |  |  | - | - | - | - | - |
| VS | 2930 | - | - | - | - | - | - | - | - | - | - | - | - |
| VS | 2932 | - | - | - | - | - | - | - | - | - | - | - | - |
| VS | 2933 | - | - | - | - | - | - | - | - | - | - | - | - |
| VS | 2934 | - | - | - | - | - | - | - | - | - | - | - | - |
| VS | 2935 | - | - | - | - | - | - | - | - | - | - | - | - |

**B**

| Group | Animal number | Viremia（10 SID_50_ FMDV challenge) | | | | | | | | | | | |
| --- | --- | --- | --- | --- | --- | --- | --- | --- | --- | --- | --- | --- | --- |
|  |  | 0 d | 0.5 d | 1 d | 2 d | 3 d | 5 d | 7 d | 9 d | 11 d | 13 d | 15 d | 17 d |
| TGCS | 18 | - | - | - | - | - | - | 616.3 | - | - | - | - | - |
| TGCS | 6 | - | - | - | - | - | - | 24279.8 | - | - | - | - | - |
| TGCS | 28 | - | - | - | - | - | - | 43.3 | - | - | - | - | - |
| TGCS | 7 | - | - | - | - | - | 122.2 | 7564.9 | - | - | - | - | - |
| TGCS | 12 | - | - | - | - | - | 21945 | 6610.9 | - | - | - | - | - |
| Non-TGCS | 24 | - | - | - | - | - | 349.9 | - | - | - | - | - | - |
| Non-TGCS | 2 | - | - | - | - | - | 84.9 | 82.1 | 88.5 | 60.2 | - | - | - |
| Non-TGCS | 4 | - | 241.5 | 224.2 | 411.3 | 1001.3 | 18293.4 | - | - | - | - | - | - |
| Non-TGCS | 3 | - | - | - | - | 35.4 | 3438 | - | - | - | - | - | - |
| Non-TGCS | 14 |  |  |  |  | 6221.7 | * | * | * | * | * | * | * |
| VS | 2922 | - | - | - | - | - | - | - | - | - | - | - | - |
| VS | 2927 | - | - | - | - | - | - | - | - | - | - | - | - |
| VS | 2928 | - | - | - | - | - | - | - | - | - | - | - | - |
| VS | 2939 | - | - | - | - | - | - | - | - | - | - | - | - |
| VS | 2929 | - | - | - | - | - | - | * | * | * | * | * | * |

Note: The number was the viral RNA copy number. “-” indicates that viremia was not detectable. “*” indicates that the pig died and samples were not collected.

**Table S2. Mutations in the FMDV sequence in the viremic serum samples after 100 SID50 (A) and 10 SID50 (B) FMDV challenge.**

**A**

| 100 SID_50_ | 2B-shRNA target region | | 3D-shRNA target region | | VP1 region | |
| --- | --- | --- | --- | --- | --- | --- |
|  | TGCS | NS | TGCS | NS | TGCS | NS |
| Mutant base ratio in the shRNA target region | 0.32% | 0.00% | 0.24% | 0.00% | * | * |
| Mutant base ratio outside of the shRNA target region | 0.07% | 0.04% | 0.15% | 0.08% | 0.17% | 0.16% |

**B**

| 10 SID_50_ | 2B-shRNA target region | | 3D-shRNA target region | | VP1 region | |
| --- | --- | --- | --- | --- | --- | --- |
|  | TGCS | NS | TGCS | NS | TGCS | NS |
| Mutant base ratio in the shRNA target region | 0.28% | 0.00% | 0.13% | 0.00% | * | * |
| Mutant base ratio outside of the shRNA target region | 0.08% | 0.14% | 0.21% | 0.11% | 0.19% | 0.09% |
